# Analgosedation and *delirium* in intensive care units in Brazil: current status. ASDUTI study

**DOI:** 10.5935/0103-507X.20180025

**Published:** 2018

**Authors:** Viviane Cordeiro Veiga, Salomón Soriano Ordinola Rojas

**Affiliations:** Unidade de Terapia Intensiva Neurológica, Hospital BP - A Beneficência Portuguesa de São Paulo - São Paulo (SP), Brasil e Comitê de Neurointensivismo, Associação de Medicina Intensiva Brasileira - São Paulo (SP), Brasil.; Comitê de Neurointensivismo, Associação de Medicina Intensiva Brasileira - São Paulo (SP), Brasil.; Unidade de Terapia Intensiva Neurológica, Hospital BP - A Beneficência Portuguesa de São Paulo - São Paulo (SP), Brasil.

**To the Editor,**

The adequate management of analgosedation and *delirium* is related to better outcomes in intensive care units.^(^^1^^-^^3^^)^ The objective of this study is to evaluate the current status of the management of analgosedation and *delirium* in Brazil.

A questionnaire was developed on the SurveyMonkey^®^ (Appendix 1) digital platform with 15 multiple-choice questions, among which some of the questions had only one option while others could have more than one option. The instrument was made available from November 2015 to February 2016. The method of selection of professionals for data collection included several multiprofessional categories.

A total of 410 professionals answered the questionnaire. Of these, 48.78% worked in public hospitals, 33.41% in private hospitals, and 17.80% in philanthropic hospitals. A total of 81.23% professionals worked in an intensive care unit (ICU) with a general profile.

The results indicated that 59.50% of the respondents used analgosedation protocols in their services, which is slightly higher than that found by Salluh et al.^(^^4^^)^ but lower than that found by Patel et al.,^(^^5^^)^ wherein 71% of the study participants used analgosedation protocols.

Among the study participants, 234 (59.24%) reported performing a systematic evaluation of pain in their ICU. Pain was evaluated up to twice a day for 35.48% of the sample, and the nurse and physician were responsible for these evaluations in most cases (51.34% and 42.78%, respectively). Sedation was evaluated up to twice a day in 49.35% of cases.

With respect to the management of sedation, validated tools (Richmond Agitation-Sedation Scale - RASS and Sedation-Agitation Scale - SAS) were used by 72.91% of the respondents, and the most commonly used sedation strategies were daily awakening (60.11%) and targeted sedation (23.94%). There was a high rate of use of validated tools (12%) compared with national data from 2009.^(^^4^^)^

The availability of analgosedation in services and its incorporation in protocols was evaluated. Midazolam, fentanyl, propofol, and morphine were the most available drugs in the analyzed institutions. The first three drugs, in addition to dexmedetomidine, were the most used in institutional protocols. The use of these drugs is common despite recommendations to avoid the use of benzodiazepines in the choice of sedative drugs.^(^^2^^)^

A total of 44.68% of the respondents evaluated *delirium* systematically. Of these, 56.72% used the Confusion Assessment Method for the ICU (CAM-ICU), and 37.37% did not use a validated tool. In the study by Patel et al., 59% of respondents systematically assessed *delirium*.^(^^5^^)^

Of the total sample, 29.62% evaluated *delirium* daily, and 23.10% performed evaluations twice a day. Physicians and nurses conducted these evaluations in 64.07% and 26.05% of cases, respectively. Haloperidol was the drug of choice for managing hyperactive *delirium* for 72.13% of participants. In addition, 12.30% of respondents used dexmedetomidine, and 11.20% used antipsychotics (Table 1).

**Table 1:** Characteristics of the assessment and management of analgosedation and *delirium*

	n (%)
Frequency of assessment of pain in the ICU	
Once daily	52 (13.37)
Twice daily	46 (11.83)
Thrice daily	78 (20.05)
Every 12 hours	40 (10.28)
Every 2 hours	54 (13.88)
Every hour	11 (2.83)
None of the above	108 (27.76)
Professional performing pain assessment	
Physician	160 (42.78)
Nurse	192 (51.34)
Physical therapist	3 (0.80)
Another professional	19 (5.08)
Frequency of assessment of sedation in the ICU	
Once daily	66 (17.14)
Twice daily	82 (21.30)
Thrice daily	69 (17.92)
Every 12 hours	42 (10.91)
Every 6 hours	33 (8.57)
Every 2 hours	29 (7.53)
Every hour	10 (2.60)
None of the above	54 (14.03)
Tool used to assess sedation	
RASS	269 (70.05)
SAS	11 (2.86)
Ramsay	78 (20.31)
None	19 (4.95)
Others	7 (1.82)
Strategy of sedation	
Daily awakening	226 (60.11)
Targeted sedation	90 (23.94)
Intermittent sedation	25 (6.65)
None	35 (9.31)
Tool used to assess *delirium*	
CAM-ICU	211 (56.72)
ICDSC	8 (2.15)
Others	14 (3.76)
None	139 (37.37)
Frequency of assessment of *delirium*	
None	121 (32.88)
Once daily	109 (29.62)
Twice daily	85 (23.10)
Thrice daily	31 (8.42)
More than thrice daily	22 (5.98)
Professional assessing *delirium*	
Physician	214 (64.07)
Nurse	87 (26.05)
Psychologist	21 (6.29)
Another professional	12 (3.59)
Drug used to manage hyperactive *delirium*	
Haloperidol	264 (72.13)
Dexmedetomidine	45 (12.30)
Antipsychotic drugs	41 (11.20)
Others	7 (1.91)
None	9 (2.46)

ICU - Intensive Care Unit; RASS - Richmond Agitation-Sedation Scale; SAS - Sedation-Agitation Scale; CAM-ICU - Confusion Assessment Method in an Intensive Care Unit; ICDSC - Intensive Care Delirium Screening Checklist.

The limitations of the study were the lack of consideration of the geographical distribution of the ICUs of respondents, lack of differentiation of responses between public, private, and philanthropic hospitals, and lack of comparison of responses among multiprofessional teams.

In conclusion, this study reveals the need to advance the routines used in the management of analgosedation and *delirium*, despite consistent evidence for improved outcomes in the literature when the recommended protocols and strategies are used.

*Viviane Cordeiro Veiga* *Neurological Intensive Care Unit, Hospital Beneficência Portuguesa de
São Paulo - São Paulo (SP), Brazil; and Neurocritical Care
Committee, Associação de Medicina Intensiva Brasileira (AMIB) -
São Paulo (SP), Brazil.* *Salomón Soriano Ordinola Rojas* *Neurological Intensive Care Unit, Hospital Beneficência Portuguesa de
São Paulo - São Paulo (SP), Brazil.*

## Appendix

**Appendix 1:** ASDUTI study questionnaire. Evaluation of analgosedation and delirium in intensive care units.

Institutional data:
Hospital profile: ( ) public ( ) private ( ) philanthropic
Unit profile: ( ) general ( ) trauma ( ) cardiologic ( ) neurological ( ) other

1. At your service, is there a protocol for analgesia and sedation?
( ) Yes
( ) No

2. Is there systematic pain assessment in your ICU?
( ) Yes
( ) No

3. How often is pain assessed at your ICU?
( ) Once daily
( ) Twice daily
( ) Thrice daily
( ) Every 12 hours
( ) Every 2 hours
( ) Every hour
( ) None of the above

4. Which professional assesses pain in your unit?
( ) Physician ( ) Nurse ( ) Physical therapist ( ) Other professional

5. Is there systematic assessment of sedation in your ICU?
( ) Yes
( ) No

6. How many times is sedation assessed in your ICU?
( ) Once daily
( ) Twice daily
( ) Thrice daily
( ) Once every 12 hours
( ) Once every 6 hours
( ) Once every 2 hours
( ) Once every hour
( ) None of the above

7. Which tool is used to evaluate sedation in your unit?
( ) Richmond Agitation-Sedation Scale (RASS)
( ) Sedation-Agitation Scale (SAS)
( ) Ramsay
( ) None
( ) Others

8. Which of these medications are AVAILABLE for use in your hospital? Check all that apply.
( ) Midazolam ( ) Morphine ( ) Fentanyl ( ) Remifentanil
( ) Sufentanil ( ) Alfentanil ( ) Dexmedetomidine ( ) Clonidine
( ) Ketamine ( ) Propofol

9. Which of these drugs are INCLUDED in your analgosedation PROTOCOL? Check all that apply.
( ) Midazolam ( ) Morphine ( ) Fentanyl ( ) Remifentanil ( ) Sufentanil
( ) Alfentanil ( ) Dexmedetomidine ( ) Clonidine ( ) Ketamine ( ) Propofol

10. Which sedation strategy do you apply in your unit?
( ) Intermittent sedation
( ) Daily awakening
( ) Targeted sedation
( ) None of the above

11. Does your unit evaluate *delirium* systematically?
( ) Yes
( ) No

12. Which tool do you use to assess *delirium*?
( ) Confusion Assessment Method in an Intensive Care Unit (CAM-ICU)
( ) Intensive Care Delirium Screening Checklist (ICDSC)
( ) Others
( ) None

13. How many times a day is *delirium* evaluated in your unit?
( ) None
( ) Once daily
( ) Twice daily
( ) Thrice daily
( ) More than thrice daily

14. Which professional assesses *delirium* in your ICU?
( ) Physician ( ) Nurse ( ) Psychologist ( ) Other professionals

15. Which drugs do you use for managing hyperactive *delirium*?
( ) Haloperidol ( ) Antipsychotics ( ) Dexmedetomidine
( ) Others ( ) None
